# Dumbbell gold nanoparticle dimer antennas with advanced optical properties

**DOI:** 10.3762/bjnano.9.205

**Published:** 2018-08-17

**Authors:** Janning F Herrmann, Christiane Höppener

**Affiliations:** 1 NanoBioPhotonics Group, Physikalisches Institut, Westfälische Wilhelms-Universität Münster, Wilhelm-Klemm-Straße 10, 48149 Münster, Germany; 2 Leibniz Institut für Photonische Technologien, Jena, Albert-Einsteinstraße 9, 07743 Jena, Germany

**Keywords:** atomistic plasmonics, dumbbell dimer antennas, electromagnetic field enhancement, light confinement, nanolens, nanoscale morphology

## Abstract

Plasmonic nanoantennas have found broad applications in the fields of photovoltaics, electroluminescence, non-linear optics and for plasmon enhanced spectroscopy and microscopy. Of particular interest are fundamental limitations beyond the dipolar approximation limit. We introduce asymmetric gold nanoparticle antennas (AuNPs) with improved optical near-field properties based on the formation of sub-nanometer size gaps, which are suitable for studying matter with high-resolution and single molecule sensitivity. These dumbbell antennas are characterized in regard to their far-field and near-field properties and are compared to similar dimer and trimer antennas with larger gap sizes. The tailoring of the gap size down to sub-nanometer length scales is based on the integration of rigid macrocyclic cucurbituril molecules. Stable dimer antennas are formed with an improved ratio of the electromagnetic field enhancement and confinement. This ratio, taken as a measure of the performance of an antenna, can even exceed that exhibited by trimer AuNP antennas composed of comparable building blocks with larger gap sizes. Fluctuations in the far-field and near-field properties are observed, which are likely caused by distinct deviations of the gap geometry arising from the faceted structure of the applied colloidal AuNPs.

## Introduction

**Figure 1:**
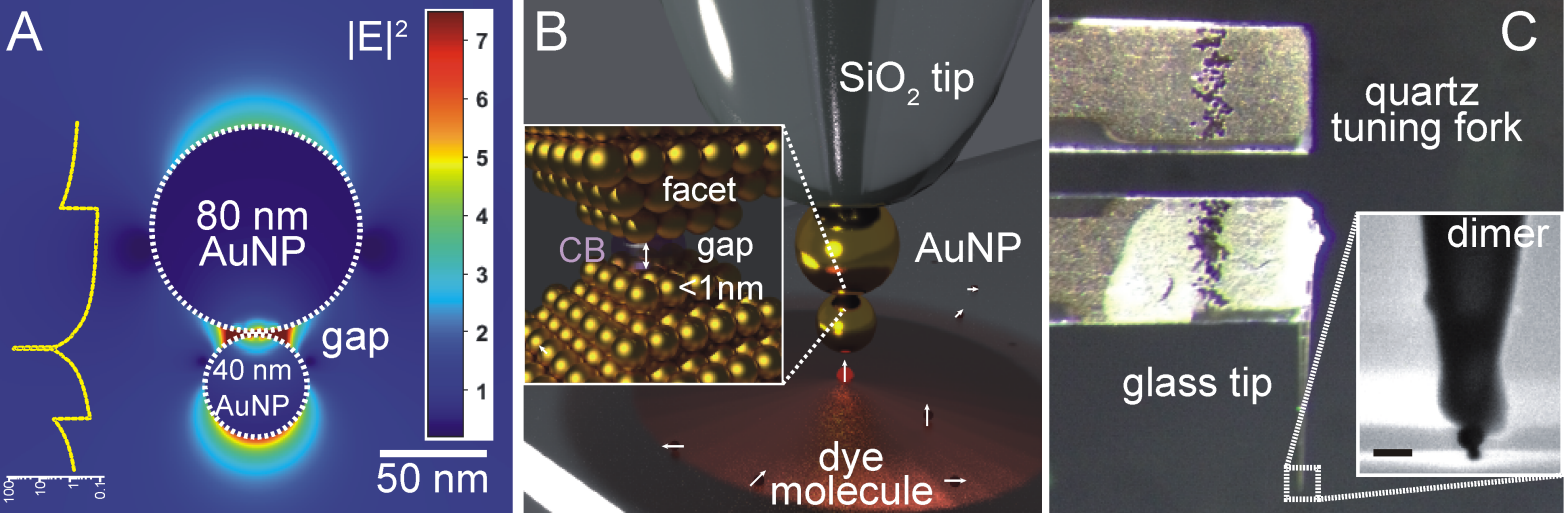
Tip-supported dumbbell antenna. (a) Calculated electromagnetic field distribution of a 80–40 nm AuNP dimer antenna with a gap size of 1 nm. (B) Schematic representation of a dimer antenna formed of spherical AuNPs attached to a sharply pointed glass tip. Inset: Magnification to the gap region showing the aligned CB[*n*]s on the NP surfaces, which results in a sub-nanometer gap distance. (C) Macroscale picture of a glass tip attached to a piezoelectric quartz tuning fork acting as a force sensor. Inset: SEM image of the CB[*n*] mediated dimer antenna attached to the pointed end of the glass tip. Scale bar: 100 nm.

The introduction of the antenna concept to the field of optics has opened up new routes to manipulate light on the nanometer scale [1–5]. For more than a decade, optical antennas have demonstrated a tremendous impact on a broad spectrum of applications [6–9]. A key function, in particular for sensing and imaging applications, is the ability of optical antennas to provide a high signal enhancement ratio and light confinement across the UV–vis–NIR spectral range. The development of new configurations has always come along with the question of fundamental limitations in regard to the obtainable electromagnetic field strength or the signal enhancement, and the achievable confinement of the light in plasmonic nanostructures [10–14]. Furthermore, this stimulated the discussion of the onset of non-classical phenomena, such as, screening effects, non-localities and charge transfer in coupled plasmonic systems [11,15–20]. Phenomena governed by non-classical physical effects have been observed already at early stages after the introduction of the concept [15,17,21], but only recently a fundamental understanding of these effects became accessible based on detailed experimental [18–19,22] and theoretical studies [23–24]. In addition to these fundamental limits, the importance of the nanoscale morphology of antennas has been identified as a key parameter affecting their far-field and near-field optical properties [25–30]. The simplest antenna geometries, whose optical response is governed by plasmonic mode coupling, are symmetric dimers formed of spherical nanoparticles. Often these structures are used as a model system to understand the impact of hot spots in more complex systems [31–32]. However, the multiplicity of modes, and with that, the ability for tailoring the plasmon resonances and the electromagnetic field distribution in these structures, is much more versatile for asymmetric (dumbbell) antennas [33–37]. Introducing a defined asymmetry for these gap structures, e.g., converts dark anti-symmetric modes into bright modes [38] and also influences non-linear responses generated in these structures [39]. In addition, the asymmetry induces a cascade of the electromagnetic field enhancement towards the pointed end of the structure such that these structures are often discussed in terms of acting as a nanolens [10,40–43]. Although the local electromagnetic field is strongest in the gap, the electromagnetic field at the end point of the smallest nanoparticle of these structures can be also increased (see for Metallic Nanopaticle Boundary Element Method (MNPBEM)-simulation of the electromagnetic field distribution [44], Figure 1A). Usually, this end point of the antenna is not considered in common SERS applications due to the one to two orders of magnitude lower electromagnetic field strength. As a consequence, the signal majorly stems from the interparticle locations. However, for applications of such dimers in TENOM or TERS, the optical response is primarily driven by the field at the end point of the probe. Theoretical investigations of the electromagnetic field distribution of these dimers at particular wavelengths demonstrate a tight connection of the optical response associated with the two particular locations, i.e., the gap and the end point [40]. Decreasing the interparticle gap size, therefore, leads to stronger electromagnetic fields at the gap and the end point locations. However, one has to keep in mind, that the reduction of the gap size also is accompanied by a shift of the plasmon resonance and the strongest electric field enhancement is observed for red-shifted excitation wavelengths.

Fabrication strategies of optical antennas, and in particular gap antennas with an optical response in the visible to NIR regime, are versatile and are often correlated with the final application schemes. Although top-down approaches, which rely on, e.g., electron-beam lithography, ion milling and other techniques, are widely used, often tailoring gap structures explores common limitations in regard to their obtainable resolution and non-invasiveness. In contrast, common bottom-up approaches often rely on the specific interaction of linker molecules, which may be used for a directed assembly of individual antenna parts. Besides of the general discussion on methodologies, the effect of the crystallinity of the employed noble metals has attracted attention [45]. The versatility of colloidal chemistry provides nowadays a tool box of nanoparticles made of different materials, shapes and sizes. In addition, the crystallinity of these structures leads to the formation of facets, edges and corners. Using colloidal nanoparticles, their chemical assembly demands for certain properties of the specific linker molecules [46]. In particular mastering the formation of sub-nanometer gap sizes requires short, rigid molecules with high chemical selectivity. In addition, these linker molecules should not affect the optical response of the formed antenna. Although sub-nanometer gap dimers can be also formed artificially by the placement of nanoparticles on a mirror substrate using defined spacer layers [47–48], in general, this approach is non-transferrable to tip-supported antennas used, e.g., in TENOM and TERS. Another frequently considered approach utilizes DNA as scaffold for the alignment of the nanoparticles [41,49–50]. In particular, DNA origami-structures provide a high versatility of the formed structures, however, the gap sizes on the sub-nanometer scale are difficult to control. Recently, we succeeded in the formation of dumbbell dimer antennas by means of the electrostatic interaction of positively charged 40 nm AuNPs and negatively charged 80 nm AuNPs [28]. Positively charged AuNPs were formed through a ligand exchange reaction with cysteamin. Incubation of a mixture of both AuNP solutions leads to the formation of AuNP dimers with gap sizes on the order of 1.3 nm to 0.8 nm. Similarly, tip-supported AuNP dimer antennas were assembled by adding a dithiol to the shell of an 80 nm AuNP, which previously has been attached to the end of a sharply pointed glass tip [10]. Due to the molecular size of the dithiol, these dimer antennas exhibit relatively large gap sizes. Commonly, the gap size is found to be on the order of 1.5–2.0 nm. Utilizing the capabilities of an AFM to manipulate such structures with sub-nanometer precision in space enables to add a smaller 40 nm AuNP to the larger one. Even though, shorter linker thiol molecules could be used instead, in principle, the high mobility of these linker molecules on the AuNP surface limits its applicability [51–52].

## Results and Discussion

### Fabrication of dumbbell dimer antennas with sub-nanometer gap size

In this study, we modify the previously established protocol of the formation of tip-supported dimer and trimer antennas by replacing the dithiol linker molecules with a rigid macrocyclic molecule (scheme, Figure 1B). Cucurbiturils (CB[*n*]s) are cyclic methylene-bridged glycoluril oligomers forming a barrel-like structure with a hollow cavity. In particular due to their sub-nanometer height of ≈0.9 nm [53], CBs can be considered as ideal spacer molecules for the formation of dumbbell dimers. Furthermore, carbonyl groups at the top and bottom face of this barrel provide a high affinity to gold surfaces. Therefore, CBs bind in a flat configuration to gold surfaces, which drives a dimer formation with well-defined gaps in terms of the gap size (inset, Figure 1B). In comparison with other linker molecules, such as dithiols, CB[*n*]-mediated dimer assembly enables to reduce the interparticle gap sizes down to the sub-nanometer regime. As a consequence, nanoparticle aggregates induced by interactions with CB[*n*]s have been shown to provide strong interparticle hot-spots, and thus, to be well-suited as SERS substrates [54–55]. The high reactivity of CB[*n*]s with Au surfaces may lead to the uncontrolled formation nanoparticle aggregates in solution, and often prevents a controlled self-assembly into dimers or small oligomer structures. However, using guided assembly by taking advantage of AFM manipulation methods, CB[*n*] is ideally suited to mediate sub-nanometer gap formation. For this, a sharply pointed glass tip is glued to a piezoelectric quartz tuning fork (Figure 1C), which enables to control the tip position with respect to AuNPs deposited on a glass surface with sub-nanometer precision in space. Briefly, the fabrication of tip-supported nanoparticle antennas includes at first the attachment of a single spherical AuNP to the pointed end of a glass tip. For this, the glass surface is functionalized with 3-aminopropoyl-trimethoxy silane (APTMS) by means of vapor deposition. Controlled interaction of the glass tip with loosely adhered Au nanoparticles on a glass surface leads to an attachment of the AuNP at the pointed end. This tip-supported AuNP is sequentially functionalized with the corresponding linker molecule, i.e., by dipping the tip-supported AuNP into an aqueous 0.5 μM CB[8] solution. After an incubation time of 5 min the tips are rinsed with milli-Q water (18 MΩ) to prevent an aggregation of CB[8] on the AuNP surface. The CB[8] modified AuNP tips are then used to attach a smaller AuNP to the tip by repeating the above described AFM-based manipulation procedure.

### Approximation of the electromagnetic field enhancement by measuring the fluorescence of high-QY emitters

Tip-supported AuNP monomers and dimers are characterized for the provided electromagnetic field enhancement and light confinement capabilities by probing the fluorescence enhancement of quantum emitters with LSPR-matched absorption and emission spectra and high intrinsic quantum yields. The latter ensures that the probed fluorescence enhancement factor stems largely from the provided electromagnetic field enhancement [1,3,56]. Since the measurements are not carried out in the regime of strong coupling, this approach only provides an approximation of the electromagnetic field strength at the end point of the dimer, since at the same time, quenching of the fluorescence occurs. Despite of this, the method provides a straight forward means to compare modifications of the electromagnetic field induced by alterations of geometrical properties [10].

**Figure 2:**
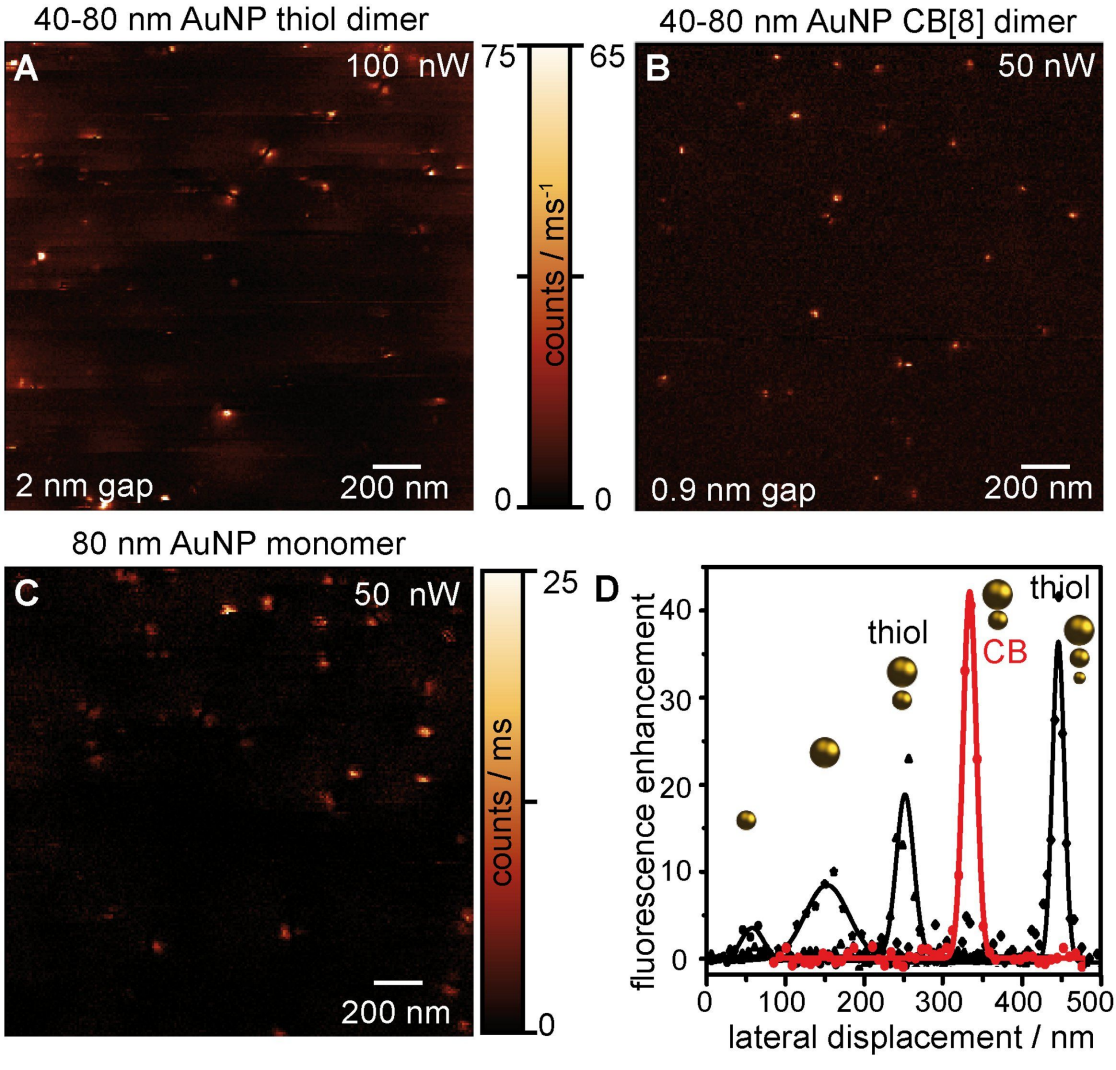
Antenna-enhanced fluorescence images of randomly distributed high-QY emitters on a glass surface imaged with dimer antennas with a gap size of 1.5 nm (A) and 1 nm (B), and a monomer antenna (C). (D) Line profiles taken from individual monomer- (40 nm and 80 nm AuNP), dimer- (40–80 nm thiol and CB[8] mediated) and trimer (20–40–80 nm thiol-mediated)-enhanced fluorescence spots normalized to the confocal background signal for comparison of the provided fluorescence enhancement and light confinement (FWHM).

Figure 2 summarizes measurements conducted with different types of monomer and dimer antennas on a high QY emitter excitable at a wavelength of λ_exc_ = 633 nm and an excitation power *P*_exc_ of 50 to 100 nW. The examples depicted in Figure 2 are typical in regard of the obtained signal strength and the noise floor. Clearly, for all measurements individual dye molecules are identified. However, the signal contrast strongly deviates for each measurement. Apparently, the best signal-to-noise level is achieved with the CB[8]-mediated dimer antenna. For comparison Figure 2C shows a fluorescence image acquired with an 80 nm monomer AuNP antenna [57–58]. The signal intensity is clearly lower than for the dimer antennas. In order to qualitatively access the light confinement and signal enhancement capabilities of these different antennas, fluorescence intensity cross-sections measured from individual spots, which can be assigned to molecules with a longitudinal orientation of the transition dipole, are comparatively displayed in Figure 2D. In addition, data points are plotted from measurements acquired with a 40 nm monomer AuNP antenna and a 80–40–20 nm trimer antenna. From these line-profiles it is obvious that the provided light confinement is largely determined by the diameter of the foremost AuNP of the antenna. Considering the radius of curvature, the light confinement is slightly better than the diameter of this AuNP, i.e., 15 nm for the 20–40–80 nm AuNP trimer antenna, ≈25 nm for the 40–80 nm AuNP dimer antennas and the 40 nm AuNP monomer antenna and 55 nm for the 80 nm AuNP monomer. Furthermore, the signal strength clearly increases with the size and number of AuNP added to the antenna structure. The latter is indicative for the expected cascade of the electromagnetic field towards the intermediate points of these gap structures. Figure 2D indicates that the CB[8] antenna provides similar light confinement capabilities than the thiol trimer. Most strikingly, the electromagnetic field enhancement of this dimer antenna reaches the same level as the more complex trimer antenna. This can be ascribed to the decreased gap size of the CB[8] dimer, which results in a stronger coupling efficiency across the gap, and thus, induces also a stronger electromagnetic field at the smallest AuNP of the dimer.

**Figure 3:**
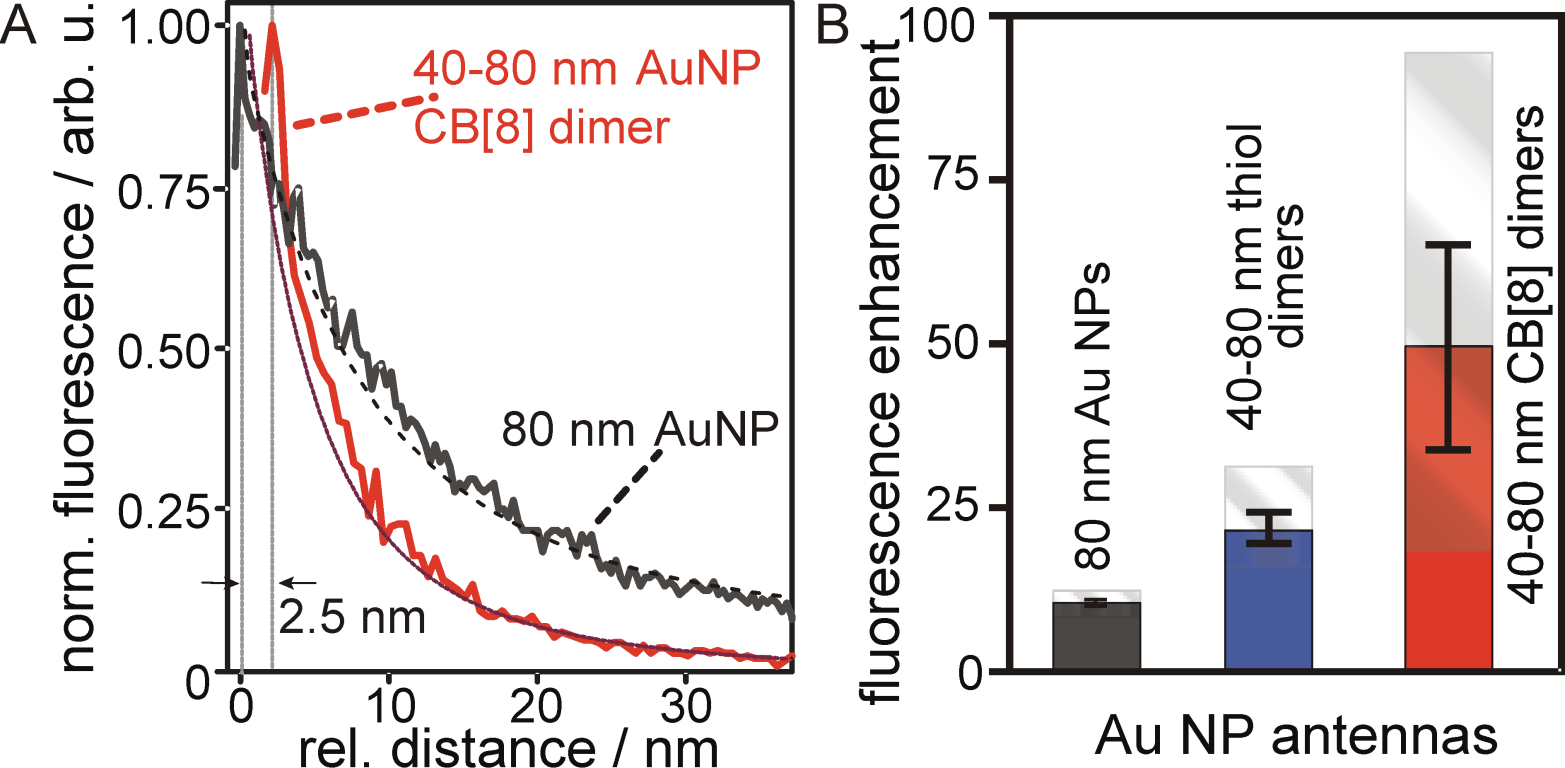
Antenna enhanced fluorescence of high QY emitters. (A) Normalized fluorescence emission rates as a function of the antenna-molecule distance for a CB[8]-mediated 40–80 nm AuNP dimer and a spherical 80 nm AuNP monomer antenna. The red, dotted and the black, dashed line correspond to the evaluation of the calculated electromagnetic field for these antennas. (B) Comparison of the absolute and averaged enhancement factors obtained for multiple 80 nm monomer antennas, thiol- and CB[8]-mediated 40–80 nm AuNP dimers.The error bars display the standard deviation.

A quantitative evaluation of the fluorescence enhancement factor can be also accomplished by measuring the fluorescence emission of single dye molecule as a function of the antenna–sample separation. The corresponding approach curves account for two mechanisms occurring when the antenna is coupled to the dye molecule: 1.) the absorption enhancement due to the interaction of the molecule with the evanescent electromagnetic field of the antenna and 2.) quenching of the excited state, i.e., the relaxation of the excited state by means of radiationless energy transfer to the metallic interface of the AuNP. Since the spontaneous emission rate equals the product of these contributions for an excitation far from the saturation limit, the corresponding approach curves exhibit a characteristic profile. Typical approach curves acquired with a monomer and a dimer antenna are displayed in Figure 3A. For antenna–sample distances larger than the corresponding diameter of the foremost AuNP, the optical response of the excited molecule corresponds to the pure confocal excitation, i.e., the evanescent field of the antenna is entirely faded out and quenching by the metallic interface of the antenna is negligible. For smaller distances at first the excitation enhancement and by reciprocity the emission enhancement are driven by the secondary field of the antenna. The exponential increase of the electromagnetic field strength with decreasing antenna–sample distance leads to a continuous increase of the emitted fluorescence signal. However, with decreasing distance, the influence of the electromagnetic field enhancement is counterbalanced by non-radiative transitions to electronic states of the metallic interface and sequential dissipation of the excited state energy. Therefore, for small AuNP-dye distances the spontaneous emission starts to decline. The onset for this decline differs clearly for the different antenna types, according to the relative distance dependence and strength of the two contributing mechanisms. Clearly, the maximum for the spontaneous emission rate mediated by the CB[8]-AuNP dimer is reached for increased distances to the molecule. Compared to the monomer antenna the onset is shifted by ≈2–2.5 nm. This offset is indicative for an increased quenching rate due to the larger total size of the dimer compared to the monomer. Despite of this increased quenching rate, the maximum electromagnetic field enhancement provided by a dimer antenna clearly exceeds the one of a simple monomer antenna with equal NP diameters. The displayed approach curves can be ideally used for determining the corresponding fluorescence enhancement factors. For this, the approach curves are corrected for the luminescence background of the antenna and the maximum spontaneous emission rate and the confocal background are evaluated. For the examples shown in Figure 3A the enhancement factor of the monomer antenna is 11 and for the CB[8]-dimer with an expected gap size of 0.9 nm the fluorescence enhancement factor yields a significantly increased value of ≈50. Taking into account that the fluorescence enhancement primarily stems from the electromagnetic field enhancement and an enhancement of the radiative rate is negligible, the decrease of the gap size clearly leads to a stronger electromagnetic field enhancement at the foremost end of the asymmetric dimer. In addition, the steep increase of the fluorescence rate within the sub-10 nm distance to the molecule demonstrates the stronger spatial light confinement provided by the dimer antenna. Taking into account this incline, it is obvious that the light confinement capabilities are dominated by the foremost, smallest AuNP of the antenna. However, a closer inspection of both approach curves also shows that for antenna-molecule separations beyond 10 nm the optical response stems largely from the 80 nm AuNP antenna.

### Plasmon resonance spectra of CB[8]-mediated dimers

Figure 3B summarizes the determined fluorescence enhancement factors for several antennas of the same kind. On average, the fluorescence enhancement factor of the 80 nm AuNP monomer antenna is 10. Clearly, the spread in the obtained maximum and minimum enhancement factors and the corresponding variance is relatively low, indicating a high reproducibility of these antennas and a low influence of deviations from the sphere geometry on the enhancement factor. The enhancement factor is further increased by utilizing the gap mode. In this case the average fluorescence enhancement factor for the thiol-mediated gap antennas is determined to ≈22 whilst the CB[8]-mediated dimers enhance the fluorescence signal of a single *z*-aligned dye molecule on average by a factor ≈48. Apparently the reproducibility for the CB[8]-mediated dimers is lower than for the thiol-mediated dimers, although a high rigidity is expected for the CB[8]. The observed fluctuations in the fluorescence enhancement likely relate to variations in their far-field optical properties. Therefore, differences in the LSPR positions, width and the amplitude of the scattering-cross section may affect the near-field optical response. Due to the small gap size of CB[8]-mediated dumbbell dimers alterations in their far-field properties from the ideal dipolar model are expected to be more pronounced than for similar thiol-mediated dimers, which possess enlarged gap sizes. Entering the regime of sub-nanometer gaps the onset of quantum effects, such as, e.g., charge screening, are known to alter the plasmon coupling, and thus, the optical response. Furthermore, antennas with sub-nanometer gaps are more sensitive to deviations from the ideal sphere geometry, gap size fluctuations and morphological changes [27].

**Figure 4:**
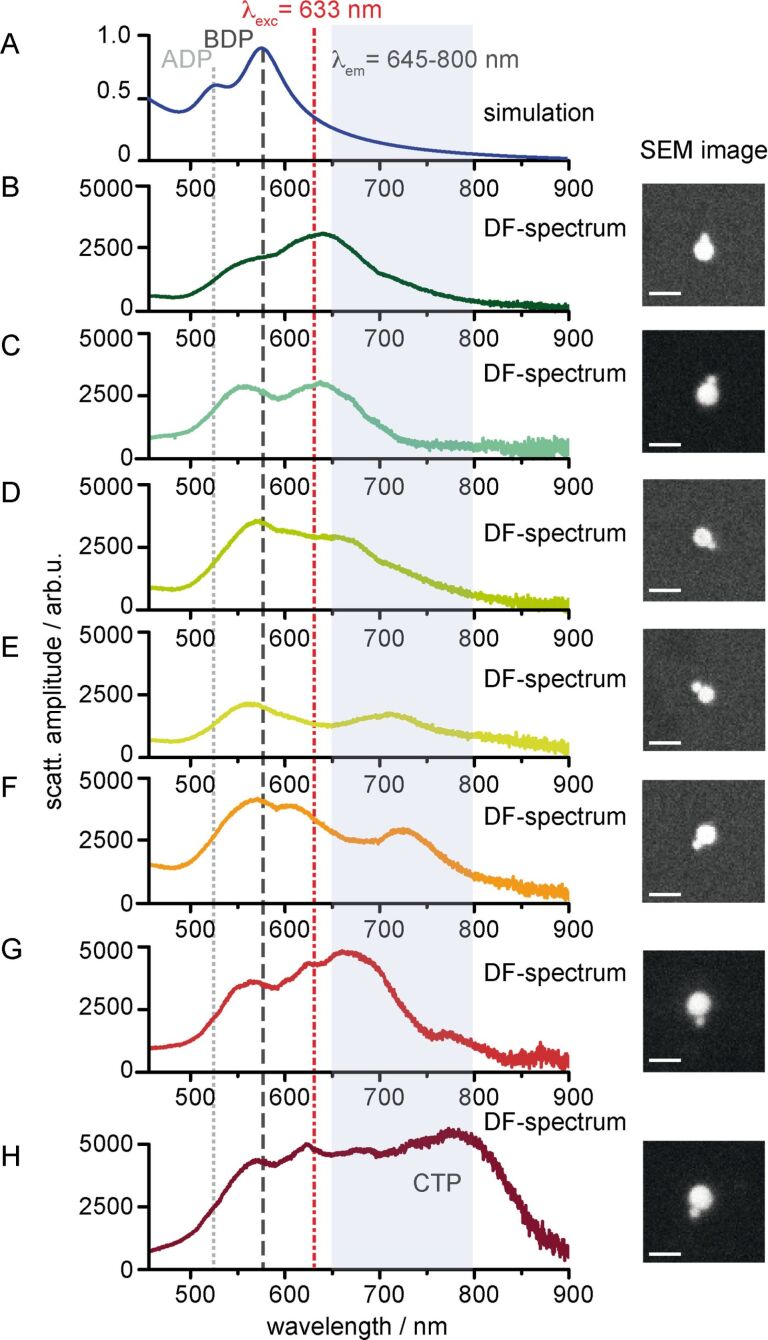
Far-field optical properties of individual CB[8]-mediated dumbbell dimers. (A) Scattering spectrum of a 40–80 nm AuNP dimer with a gap size of 1.0 nm calculated within the dipolar approximation limit. (B–H) Dark-field transmission spectra of individual dimers assembled in solution and deposited on a carbon-coated TEM grid. Insets: SEM images of the corresponding dimers (Scale bar: 100 nm).

Therefore, dark-field spectra are recorded of individual CB[8]-mediated dimers, which are compared to the calculated scattering spectrum of an idealized dimer with a gap size of 1.0 nm (Figure 4). Clearly, the scattering spectrum calculated within the dipolar approximation limit reveals the dipolar bonding dimer plasmon (BDP) mode at 572 nm, and blue-shifted from the BDP, also the dipolar anti-bonding dimer plasmon (ADP) mode (Figure 4A), which is characteristic for the asymmetric dimer geometry. The experimentally recorded dark-field spectra displayed in Figure 4B–Figure 4H reflect this profile. However, the BDP and ADP peak positions are red-shifted and broadened in comparison to the calculated spectrum (Figure 4A). This can be largely explained by the different environmental conditions, i.e., the calculation does not account for the thin carbon substrate used for the measurements.

In addition to the dipolar mode structure, multiple spectra are characterized by a red-shift and a broadening of the BDP mode or even exhibit an additional peak (CTP-charge transfer plasmon mode) in the NIR region. The latter is indicative for the onset of charge transfer mechanisms. The origin for these deviations is not evident from the geometrical structure of the investigated dimers. Both, the size and the shape of the dimer-forming AuNPs appear relatively homogeneous from the additionally displayed SEM images (Figure 4). Furthermore, strong size variations can be also excluded for the tip-supported dimers based on the height information collected during the attachment process, the observed light confinement and the post-characterization by means of scanning electron microscopy imaging (SEM image, inset Figure 1C).

Since the spontaneous emission rate is taken as a measure for the electromagnetic field enhancement, the influence of the observed changes in the dark-field (DF) scattering spectra on the emission rate has to be considered. As outlined before, the spontaneous emission rate depends on the excitation/emission rate γ_exc/em_ enhancement and on the quenching rate γ_abs_. Despite of the origin of the observed spectral fluctuations of the individual dimers, these impose strong consequences on the fluorescence enhancement capabilities of a dimer antenna, since the modification of the corresponding molecular transition rates is obeyed by different spectral dependencies. This usually finds evidence in the observation, that the fluorescence enhancement is strongest red-shifted from the LSPR peak. Taking into account the excitation wavelength of λ_exc_ = 632.8 nm used for the characterization of near-field optical properties of the dimers, the excitation rate enhancement is strongest for the dimers shown in Figure 4F–Figure 4H. For these dimers the scattering amplitude at λ_exc_ is approximately twice as strong as for the most regular spectra (Figure 4B and Figure 4C). Furthermore, these dimers possess also a strong scattering cross-section across the spectral emission region. Finally, the spectral resonance of the quenching rate has also to be taken into account additionally, which is usually blue-shifted from the LSPR. As a consequence of these dependencies, the effective local density of states (LDOS) varies.

**Figure 5:**
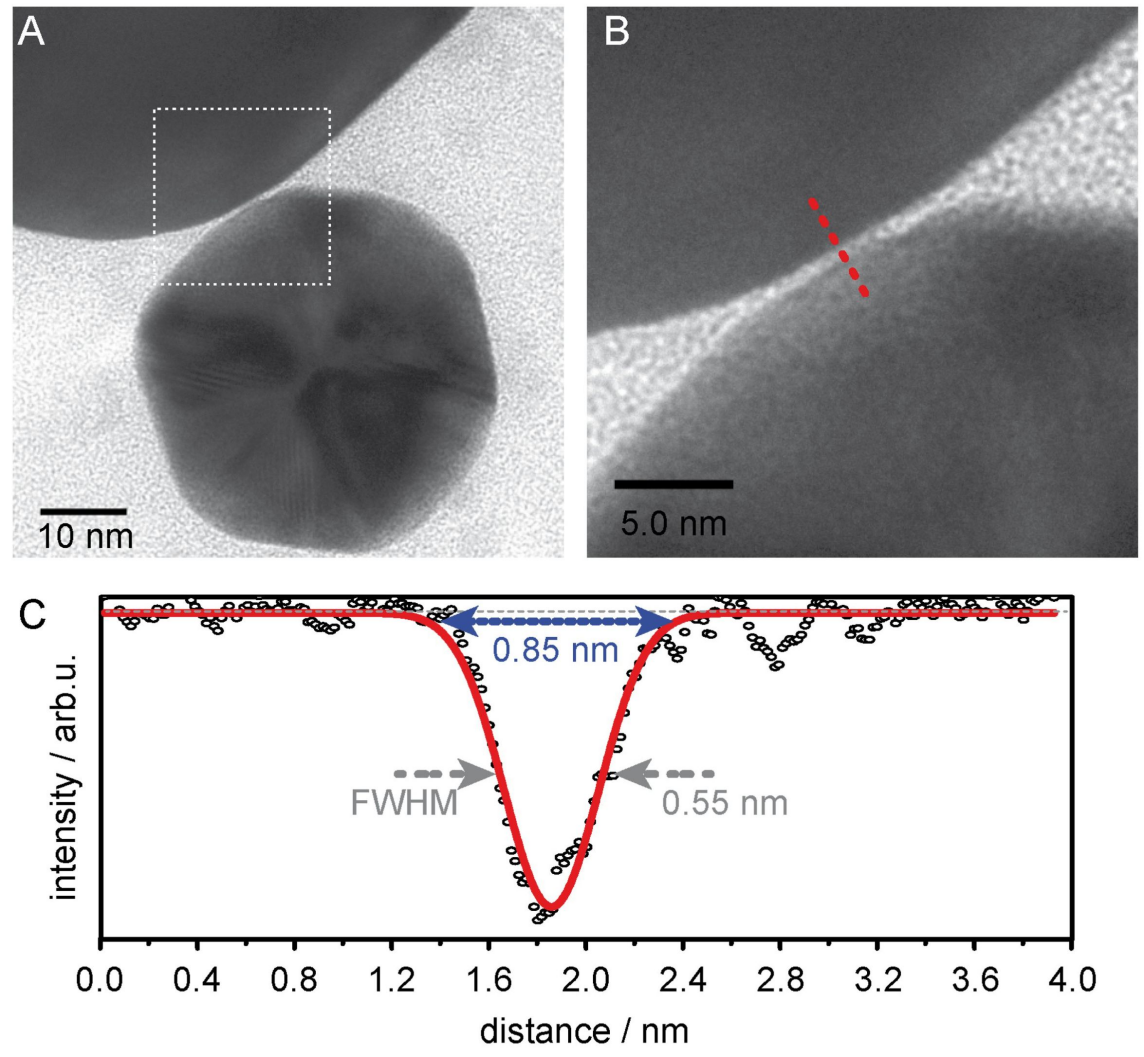
Gap size of CB[8] mediated 40–80 nm AuNP dimers. (A) TEM image of a dimer with sub-nanometer gap size. (B) High-magnification TEM image of the gap region. (C) Line-profile drawn across the gap (red dotted line in B).

The observed spectral variations for geometrically similar dimers are likely correlated to the spread in the fluorescence enhancement factor. In principle, these observed variations in the far-field and near-field optical properties may have different origins. Particularly, the particle size, particle geometry and gap capacitor properties, such as, the gap size, the gap conductivity and the gap morphology are the most intuitive parameters, which have been shown to affect the LSPR position, width and scattering cross-section [18,25,28,36,59–60]. As a first parameter, variations in the gap size have to be considered. Figure 5A and Figure 5B display TEM images of a typical dumbbell dimer with a sub-nanometer gap size. In accordance with the used CB[8] linker molecule a gap size of 0.85 nm is found (lineprofile, Figure 5C). Overall, the observed gap sizes are in excellent agreement with the known height of CB[8] and only minor deviations occur.

### Nanoscale morphology of the gap region

**Figure 6:**
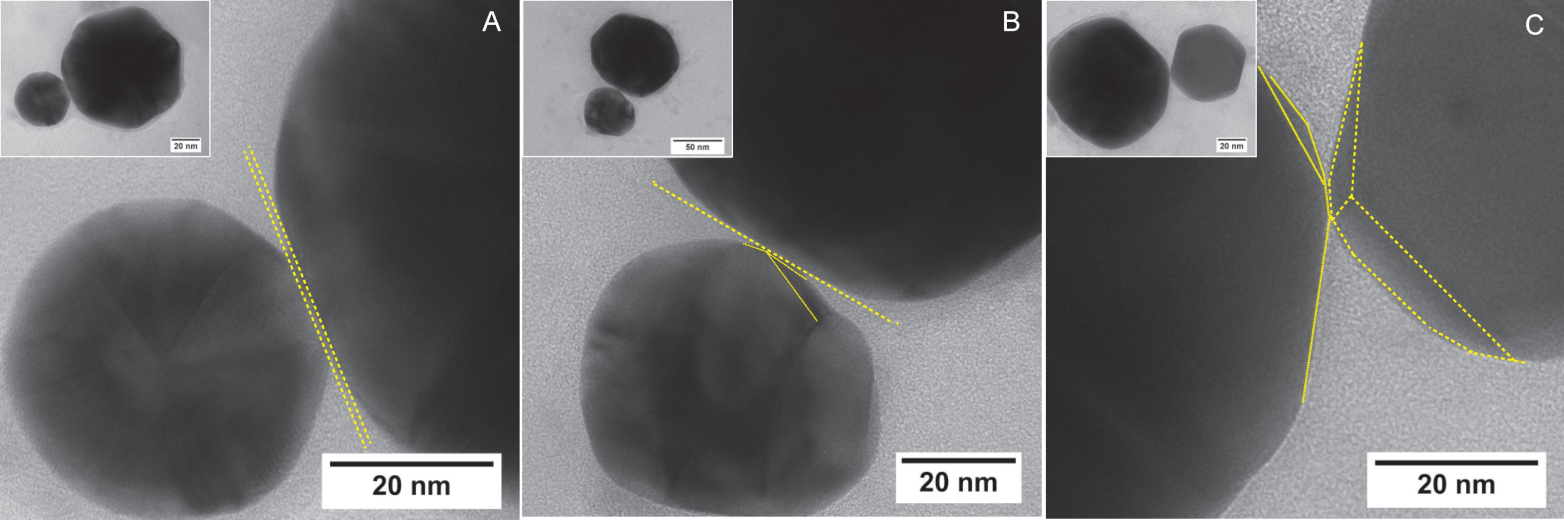
Morphologies of dumbbell dimers with sub-nanometer gap sizes. (A) Dimer with a planar gap. (B) Dimer with a gap formed by a large facet of the 80 nm AuNP and edge of the 40 nm AuNP. (C) Dimer with a pointed gap morphology formed by the interaction of the facet edges/corners of both contributing AuNPs.

Furthermore, taking into account size variations of the dimer-forming AuNPs, these correlate well with the variations observed for the thiol-mediated dimers, which provide a relatively uniform enhancement factor. Therefore, minor geometric deviations are likely to be ruled out as a source for the wide spreading of enhancement factors found for the CB-mediated dimers. Therefore, dimers with sub-nanometer gaps are in addition characterized for their nanoscale structure in the gap region. High resolution TEM images of the formed dimers immediately reveal the faceted surface of the AuNPs. Therefore, these NPs do not match with the shape of a perfect sphere used frequently for the simulations. Recently, it has been shown that the uniformity and size of the facets has strong influence on the homogeneity of their LSPR spectra [28,30,60]. From the high-magnification TEM images of the gap region of the dimers displayed in Figure 6, it is obvious that the facet-nature of the AuNPs leads to multiple possible gap configurations. Large planar gaps are formed in the case that the two AuNPs assemble in a fashion that the gap-forming facets are aligned parallel to each other (Figure 6A). However, the gap formation can include also facet edges (Figure 6B) or the corner of several facets merging in this point (Figure 6C). In this case the gap morphology changes from a larger planar gap into a smaller pointed gap, which modifies the mode volume, and thus, can lead to a stronger light concentration in the gap region, and thus, results in a higher electromagnetic field strength [61]. The facet edges, corners and atomic protrusion can be considered as points where the electromagnetic field is strongly confined, i.e., they can serve as hot spots of the electromagnetic field [14]. Therefore, the gap morphology of sub-nanometer size gaps has attracted high attention and recent investigations have shown a strong influence on the far-field properties of such gap antennas. For the CB[8]-mediated dimers with sub-nanometer gap sizes it is likely that different gap morphologies can alter the electromagnetic field confined to the end-point of the smallest sphere, especially if onsetting quantum effects lead to a redistribution of the electromagnetic near-field.

## Conclusion

In conclusion, CB[8]-mediated dumbbell dimers formed of 40 nm and 80 nm AuNP dimers are shown to exhibit advanced properties in regard to the signal enhancement ratio and light confinement as compared to AuNP dimers with gap sizes of twice that of CB[8]-dimers. In particular, it is demonstrated that the enhancement of the electromagnetic field of the CB[8]-dimers is comparable to that of a similar trimer antenna composed of 20 nm, 40 nm and 80 nm AuNPs. In a few instances, even higher enhancement factors have been observed. Although the gap size reduction explains well the higher electromagnetic field strength at the end point of the dimer, the broad variation of enhancement factors assigned to multiple dumbbell dimers cannot be assigned to gap size fluctuations. The rigidity of CB[8] compared to alkane thiol linker molecules should lead to a high stability of the gap size. TEM images reveal gap sizes of ≈0.85 nm. In addition to fluctuations in the electromagnetic field enhancement, strong variations in their far-field response are observed, which influence the near-field response in terms of the enhancement of the fluorescence emission of a single quantum emitter. The deviations are likely correlated to different gap configurations, which are easily identified in HR-TEM studies. The influence of the gap morphology on the optical properties turns out to be non-negligible. However, this effect becomes only significant for sub-nanometer size gaps. As such, the observed deviations are likely also to be governed by a modified onset of quantum size effects.

## Methods

### Fluorescence measurements

All optical measurements are acquired with an in-house built microscope, which combines a confocal microscope with an AFM setup for tip-enhanced fluorescence measurements. A detailed description of the microscope can be found in [62]. Briefly, the system uses a linearly polarized 15 mW HeNe laser (λ_em_ = 632.8 nm) as an excitation source for the fluorescence measurements. The laser beam is converted into a radially polarized beam by means of a liquid crystal mode converter (ArcOptics, Switzerland) which is coupled to an inverse microscope (Ti-U, Nikon, Japan) and is tightly focussed with a high NA objective (100× Plan APO, NA 1.49, Nikon, Japan) to the tip–sample region. The emitted fluorescence signal is collected with the same objective and is spectrally filtered for discrimination from the excitation light by means of a combination of dichroic mirrors and bandpass filters. The signal is detected by means of an Avalanche photodiode (SPCM-AQRH-TR, Excelitas, Canada). Images are recorded by scanning the sample through the laser focus using a piezoelectric scanner (Nano-H, Mad City Labs, USA). For each image pixel the emitted fluorescence signal is integrated for 5–10 ms. For the antenna-enhanced fluorescence emission, the antenna is precisely aligned in the laser focus and the antenna–sample distance is maintained with sub-nanometer accuracy to 2–4 nm by means of a force feedback loop regulating on the frequency shift of the force sensor, which is excited at its resonance frequency. The fluorescence emission rate as a function of the antenna–sample distance is recorded with the feedback loop switched off, and retracting the antenna by 50 nm from the surface. The high quantum yield dyes Alexa633 and Alexa680 respectively, are applied for these measurements, which provide suitable absorption and emission properties in regard to the LSPR of the applied monomer and dimer AuNP antennas.

### Dark-field spectroscopy

Dark-field spectra are acquired using a commercial dark-field condensor (TI-DF-NA 1.45-1.2, Nikon, Japan) for white light illumination. The scattered light is collected with a 100× objective with a variable numerical aperture, which is adjusted to ≈0.6, and spectrally resolved by means of a spectrograph with coupled CCD camera (Shamrock-303i-A/Newton EMCCD, Andor, Ireland). The recorded spectra are corrected for the backgroung and for the spectrally varying detection efficiency of the CCD chip.

### SEM/TEM investigations

SEM images are recorded with a Zeiss Gemini Crossbeam FIB/SEM with an acceleration voltage of 5 kV and the TEM investigations are carried out with a a FEI Technai G^2^20 with an acceleration voltage of 200 kV, respectively, in brightfield mode. In order to access the correct gap size of the asymmetric dimers the TEM stage is tilted in 0.1° steps. TEM images with a high magnification used for the determination of the gap size are acquired for the tilt position providing the largest gap size.
